# Perirenal Adipose Tissue in Cardiovascular Disease: From Molecular Insights to Therapeutic Perspectives

**DOI:** 10.3390/biomedicines14081804

**Published:** 2026-08-11

**Authors:** Adriana Grigoraș, Rodica Radu, Andrei Prodaniuc, Florin Dumitru Petrariu, Viorel Dragoș Radu, Cornelia Amalinei

**Affiliations:** 1 Department of Morphofunctional Sciences I, Grigore T. Popa University of Medicine and Pharmacy Iasi, 700115 Iasi, Romania; andreiprodaniuc@yahoo.com (A.P.); cornelia.amalinei@umfiasi.ro (C.A.); 2 Department of Histopathology, Institute of Legal Medicine, 700455 Iasi, Romania; 3 Internal Medicine Department, Grigore T. Popa University of Medicine and Pharmacy Iasi, 700115 Iasi, Romania; rodica.radu@umfiasi.ro; 4 Department of Preventive Medicine and Interdisciplinarity, Grigore T. Popa University of Medicine and Pharmacy Iasi, 700115 Iasi, Romania; florin.petrariu@umfiasi.ro; 5 Department of Urology, Grigore T. Popa University of Medicine and Pharmacy Iasi, 700115 Iasi, Romania

**Keywords:** perirenal adipose tissue, obesity, cardiovascular diseases, hypertension, atherosclerosis, adipokines

## Abstract

Perirenal adipose tissue (PRAT) has emerged as a clinically relevant endocrine organ connecting obesity to cardiovascular disease (CVD), chronic kidney disease, and certain malignancies. Its unique anatomical location, surrounding the kidneys, accounts for PRAT’s role in altering intrarenal haemodynamics and hydrostatic pressure. Accordingly, PRAT’s expansion is associated with the activation of the renin–angiotensin–aldosterone system (RAAS), further increasing blood pressure. Adipokine dysregulation, together with overexpression of miR-24-3p, miR-155, miR-146a, and miR-21 in PRAT, modulates inflammation and oxidative stress, leading to endothelial dysfunction and increased risk of atherosclerosis and hypertension in obesity. Imaging assessment of PRAT thickness through computed tomography, magnetic resonance, or ultrasound has also emerged as a complementary measure for the evaluation of CVD risk. Potential therapeutic strategies targeting PRAT include lifestyle interventions, antidiabetic agents, RAAS inhibitors, adipose tissue browning agents, NOD-like receptor protein 3 (NLRP3) inflammasome inhibitors, peroxisome proliferator-activated receptor gamma (PPARγ) agonists, and surgery. Currently, novel therapeutic interventions targeting PRAT activity in CVD, such as senotherapeutic strategies, bioengineering approaches aimed at enhancing adipose-derived mesenchymal stem cell (ADMSC) function, gut microbiota modulation, and colchicine and bone morphogenetic protein 4 (BMP4) administration, are also being explored. In light of these findings, PRAT’s clinical relevance extends beyond its energy storage role, highlighting it as a metabolically active fat depot. Its assessment and therapeutic modulation may complement existing cardiovascular prevention strategies, particularly in patients with obesity.

## 1. Introduction

Located within the retroperitoneal space, perirenal adipose tissue (PRAT) is a distinct visceral adipose tissue depot that surrounds the kidneys. Due to its unique anatomical location and distinctive morphology, PRAT has recently attracted increasing clinical interest as a potential therapeutic target for the prevention and management of various conditions, including cardiovascular diseases (CVDs) in patients with obesity [1]. Moreover, considering its variable composition, particularly related to the abundance of brown adipocytes, imaging-based assessment of PRAT thickness in obesity has consistently emerged in recent years as a valuable tool for the prognostic stratification of metabolic dysfunction associated with different pathologies, including malignancies, chronic kidney disease, hypertension, and atherosclerotic cardiovascular disease (ASCVD) [2,3,4,5,6].

PRAT displays a physiological contribution to metabolic and vascular homeostasis by releasing a panel of adipokines and cytokines, such as leptin, adiponectin, visfatin, resistin, IL-1β, IL-6, and tumour necrosis factor-α (TNF-α) [7]. These factors modulate glomerular filtration, vascular tone, endothelial function, and blood pressure through their paracrine and endocrine actions, supporting cardiovascular and renal homeostasis [4,7].

PRAT dysfunction disrupts the balance between protective adipokines and pro-inflammatory cytokines, contributing to endothelial dysfunction, lipotoxicity, systemic chronic inflammation, atherosclerotic plaque progression, and hypertension in obesity [1,8,9,10]. Moreover, PRAT expansion has been associated with the activation of both the sympathetic nervous system and the renin–angiotensin–aldosterone system (RAAS), mechanisms that may further increase cardiovascular risk in patients with obesity [5,11,12]. Increased PRAT mass may also induce local hypoxia, which promotes the activation of inflammatory cascades characterised by increased secretion of adipocyte-derived pro-inflammatory cytokines and chemokines. These mediators further induce adipocyte dysfunction and facilitate immune-cell infiltration into the PRAT area [13].

Obesity-associated chronic low-grade inflammation in PRAT may also exacerbate oxidative stress and alter adipocyte activity, leading to the impairment of endothelial nitric oxide (NO) homeostasis, a master regulator of vascular tone. Endothelial dysfunction may subsequently induce vascular smooth-muscle-cell proliferation, NOD-like receptor protein 3 (NLRP3) inflammasome activation, and increased vascular tone, contributing to CVD development [12].

Based on current knowledge, strategies aimed at preventing cardiovascular events by targeting PRAT include lifestyle interventions, as well as pharmacological therapies and surgical approaches designed to decrease adipose tissue accumulation [11,13]. In this regard, accumulating evidence supports the protective effects of different therapeutic agents, including sodium-glucose cotransporter 2 (SGLT2) inhibitors, niacin, adipose tissue browning agents, peroxisome proliferator-activated receptor gamma (PPARγ) agonists, glucagon-like peptide-1 receptor agonists (GLP-1 RAs), RAAS inhibitors, and NLRP3 inflammasome inhibitors [4,9,10,13,14,15,16]. In addition to these approaches, several emerging therapeutic strategies have shown potential for CVD management. These include modulation of bone morphogenetic protein 4 (BMP4) expression, suppression of PRAT inflammation through colchicine administration, modulation of the gut microbiota, senotherapeutic interventions targeting the accumulation and deleterious effects of senescent cells within PRAT, and tissue engineering approaches aimed at enhancing the regenerative and therapeutic potential of adipose-derived mesenchymal stem cells (ADMSCs) [10,17,18,19,20]. At the same time, integration of deep learning algorithms with magnetic resonance imaging (MRI)-based assessment of PRAT thickness may improve cardiovascular risk stratification and facilitate the early diagnosis of CVD in patients with obesity [21,22].

While epicardial adipose tissue (EAT) and PRAT are ectopic fat depots associated with increased cardiovascular risk, they provide complementary information, while EAT primarily mirrors local inflammatory and coronary atherosclerotic processes, whereas PRAT represents a marker of the cardio–renal–metabolic axis, reflecting systemic pathways underlying the association between visceral adipose tissue, renal dysfunction, and cardiovascular disease. Therefore, PRAT assessment may provide additional value for cardiovascular risk stratification in obesity, although additional prospective studies are required to establish its independent prognostic significance and clinical applicability.

Although significant progress has been made in recent decades in deciphering the molecular mechanisms linking PRAT dysfunction in obesity to increased cardiovascular risk, the precise pathways involved in these processes and their therapeutic implications remain partially understood. This may be attributed to the unique structural features of the PRAT depot, its morphological variability associated with age and gender, and its functional relationship with renal function [12,23]. In this context, this review aims to elucidate the role of PRAT and the molecular pathways linking its dysfunction to CVD, providing novel therapeutic opportunities in patients with obesity. Moreover, by integrating the detailed morphological characterization with molecular insights, added to recent advances in PRAT biology, including exosome-mediated intercellular communication, along with the identification of novel molecular pathways and innovative PRAT-targeted therapeutic strategies, this study offers a comprehensive and up-to-date perspective on the contribution of PRAT dysfunction to obesity-associated CVD.

## 2. Perirenal Adipose Tissue-Specific Location and Structure

Adipose tissue, conventionally classified into subcutaneous and visceral fat depots, represents one of the largest and most metabolically active organs in the human body. Beyond its fundamental role in lipid storage and energy homeostasis, it exerts pleiotropic endocrine functions by secreting a large spectrum of adipokines and cytokines. Thus, it contributes to the pathogenesis of multiple disorders, including CVD, chronic kidney disease, type 2 diabetes mellitus (T2DM), depression, and different malignancies [3,7,24].

As a component of visceral adipose tissue, PRAT is anatomically situated between the renal capsule and Gerota’s fascia. This area has emerged as a fat depot of particular physiological relevance, providing mechanical support for the kidneys, in addition to playing a significant role in local and systemic homeostasis. PRAT surrounds each kidney and its ipsilateral adrenal gland. It receives its vascular supply from multiple branches of the abdominal aorta, has predominantly sympathetic autonomic innervation, and its lymphatic drainage is achieved through the para-aortic lymphatic system [7]. PRAT is also characterised by a higher proportion of saturated fatty acids compared to subcutaneous adipose tissue, a feature associated with increased inflammatory activity and adverse cardiometabolic effects [7]. Similar to other visceral adipose depots, this fat tissue originates from preadipocytes that lack the expression of endothelial markers (e.g., CD31) and haematopoietic stem cell markers (i.e., CD45) but retain CD90 and CD166 expression [2,25].

From a morphological perspective, PRAT presents a heterogeneous cellular composition comprising white and brown adipocytes, with relative proportions dynamically changing throughout life [12]. In addition, it contains a broad cellular spectrum, including preadipocytes, mesenchymal cells, endothelial cells, pericytes, and a low amount of immune cells, along with numerous capillaries and nerve endings [7]. PRAT activity is determined not only by its volume but also by its cellular composition and tissue architecture, including the abundance of brown adipocytes, the number and activation state of resident immune cells, and the extent of sympathetic innervation. These features collectively regulate the secretory profile of PRAT and modulate its contribution to CVD development.

**Figure 1:**
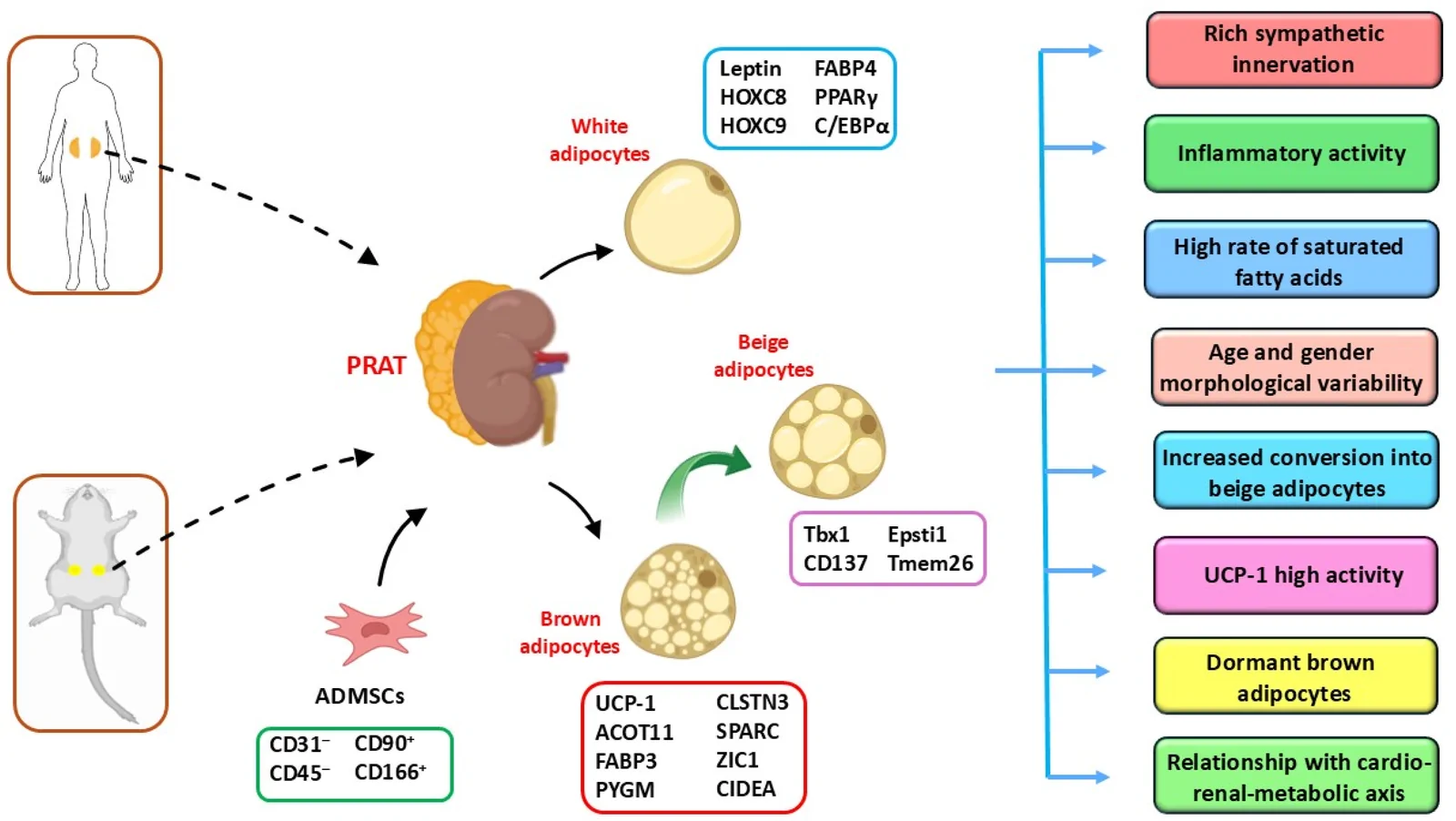
The morphological architecture of PRAT. PRAT is a visceral adipose tissue depot located around the kidneys. In adults, PRAT is primarily composed of white and brown adipocytes, which can be identified by their specific molecular markers. PRAT adipocytes are derived from ADMSCs, which express CD90 and CD166 but lack the expression of CD31 and CD45. In obesity, PRAT undergoes a brown-to-white adipocyte transition, accompanied by the appearance of beige adipocytes expressing markers such as Tbx1, CD137, Epsti1, and Tmem26. Overall, PRAT contains a relatively high proportion of dormant brown adipocytes, is richly innervated by sympathetic nerves, exhibits high levels of saturated free fatty acids, and plays an important role in the cardio–renal–metabolic axis. ACOT11—acyl-CoA thioesterase 11; ADMSCs—adipose-derived mesenchymal stem cells; C/EBP α—CCAAT-enhancer-binding protein α; CIDEA—cell death-inducing DFFA-like effector a; CD—cluster of differentiation; CLSTN3—calsyntenin-3; Epstil1—epithelial stromal interaction 1; FABP—fatty acid-binding protein; HOX—homeobox; PPARɣ—peroxisome proliferator-activated receptor ɣ; PRAT—perirenal adipose tissue; SPARC—secreted protein, acidic, and rich in cysteine; Tbx1—T-box protein 1; Tmem26—transmembrane protein 26; UCP-1—uncoupling protein 1; ZIC1—Zic family member 1.

PRAT is predominantly composed of brown adipocytes, while white adipocytes form only a thin peripheral layer during intrauterine development and the first year of postnatal life [7]. Thereafter, the tissue undergoes marked morphological and functional remodelling. Thus, white adipocytes constitute the predominant cellular population in adulthood. In contrast, brown adipocytes, exhibiting a multilocular feature due to the presence of multiple lipid droplets and high levels of metabolic activity, are confined to discrete clusters, mostly located in regions rich in sympathetic nerve endings [7]. Importantly, most brown adipocytes of adult PRAT exhibit a dormant phenotype characterised by elevated uncoupling protein 1 (*UCP-1*) expression and unilocular morphology, as well as the expression of acyl-CoA thioesterase 11 (*ACOT11*); fatty acid binding protein 3 (*FABP3*); glycogen phosphorylase, muscle-associated (*PYGM*); calsyntenin-3 (*CLSTN3*); and secreted protein acidic and rich in cysteine (*SPARC*) (Figure 1) [25,26]. Furthermore, morphological studies of adult PRAT have revealed that its central region, located around the renal hilum, is a form of visceral brown adipose tissue, owing to the high abundance of UCP-1-expressing brown adipocytes, while its lateral part is predominantly composed of white adipocytes [27]. However, PRAT brown-to-white conversion, or whitening, which accompanies advancing age, may nonetheless be modulated by a range of environmental factors (i.e., chronic exposure to low temperatures). For instance, autopsy-derived PRAT samples from individuals residing in Siberia display up to 40% brown adipocytes [28]. At the molecular level, UCP-1 expression in white adipocytes promotes their differentiation into beige adipocytes, which exhibit intermediate morphological features between white and brown adipocytes and exhibits thermogenic responsiveness to cold exposure [26]. This phenotypic transition is also characterised by overexpression of T-box protein 1 (*Tbx1*), *CD137*, epithelial stromal interaction 1 (*Epsti1*), and transmembrane protein 26 (*Tmem26*), along with decreased expression of classical white adipocyte markers, including homeobox protein Hox-C 8 (*HOXC8*), *HOXC9*, and *leptin* (Figure 1) [26].

PRAT also exhibits gender-based morphological variability, with a significantly higher rate of brown-like adipocytes expressing UCP-1 observed in women compared with men (33% vs. 7%) [23]. Moreover, PRAT demonstrates a higher predisposition to the acquisition of brown adipocyte-like features in women, a feature that may be partially attributed to Y-chromosome-mediated suppression of UCP-1 expression [2].

PRAT is also characterised by a relatively low immune-cell content, mainly represented by macrophages and mast cells, under physiological conditions [29,30]. However, aging is associated with the acquisition of a highly immunogenic PRAT milieu, accompanied by enhanced T-cell responses, potentially driven by increased infiltration of natural killer (NK) cells within this fat area [29]. Furthermore, age-related remodelling of the stromal vascular fraction of the perirenal adipose tissue (PRAT-SVF) compartment is characterised by increased abundances of macrophages, NK cells, and T lymphocytes, in association with a decline in stromal-cell content [30].

Collectively, PRAT should be considered a dynamic visceral adipose tissue with a distinct morphological architecture that varies throughout life and is capable of adapting to microenvironmental conditions. However, comprehensive morphological and molecular characterization of the PRAT microenvironment is limited due to the surgical procedures required for tissue acquisition, restricting the availability of human samples for investigation. Physiological structural remodelling of PRAT may significantly affect both local and systemic homeostasis, mainly through changes in adipocyte morphology and immune-cell content. The acquisition of a pro-inflammatory PRAT immune phenotype may further increase cardiovascular risk in older people.

## 3. Imaging Assessment of PRAT in Cardiovascular Diseases

In recent years, the imaging assessment of PRAT thickness by ultrasound, magnetic resonance imaging or computed tomography (CT) has emerged as an important tool in the evaluation of cardiovascular risk, mainly among patients with obesity and/or T2DM [7,31]. Furthermore, PRAT thickness appears to correlate positively with waist circumference and body mass index (BMI), suggesting that it may serve as a useful marker for the evaluation of visceral adiposity and cardiovascular risk prediction [2,12].

Mean PRAT thickness is 7.95 mm in healthy individuals [32]. Gender-related differences have also been reported in CT assessments, with mean PRAT thickness values of 5 ± 2 mm in women and 8 ± 2 mm in men among normal subjects [33]. Moreover, PRAT thickness exceeding 10 mm or a PRAT area greater than 100 cm^2^ is considered suggestive of abnormal fat depot accumulation [7], whereas PRAT thickness may reach up to 26.54 mm in patients with obesity [32].

Evidence from recent decades has revealed that PRAT expansion may be involved in increasing the risk of cardiovascular and/or metabolic diseases, especially among patients with obesity [7,12,31]. In this context, a recent study conducted in a large cohort of living kidney donors showed that hypertensive patients have significantly increased lateral and posterior PRAT thickness in CT compared to their counterparts in normotensive individuals (13.2 mm vs. 9.8 mm and 8.5 mm vs. 5.2 mm, respectively) [34]. Moreover, PRAT thickness was significantly higher in patients with obesity and hypertension compared to non-hypertensive individuals (13.6 ± 4.8 mm vs. 11.6 ± 4.1 mm) [11]. Concurrently, in the same study, a significant correlation between PRAT thickness and age, BMI, waist circumference, creatinine, serum insulin level, glycated haemoglobin, and homeostatic model assessment for insulin resistance (HOMA-IR) was reported [11]. Similarly, a study conducted on 166 individuals with high–normal blood pressure demonstrated that obesity-related parameters (waist circumference and BMI), together with metabolic biomarkers (serum uric acid, triglyceride–glucose index, fasting plasma glucose, and urinary albumin-to-creatinine ratio) were significantly higher in patients with an ultrasound-assessed PRAT thickness >30 mm compared with patients with PRAT thickness ≤30 mm [22]. According to the same study, a PRAT expansion over 35.5 mm also demonstrated moderate predictive accuracy for incident hypertension in individuals with high–normal blood pressure, reaching 75% specificity and 68% sensitivity [22]. Consequently, careful blood pressure monitoring in normotensive individuals who exhibit PRAT expansion may facilitate the early detection of hypertension.

A recent cross-sectional study also showed that CT-assessed PRAT expansion is independently associated with alterations of circadian blood pressure patterns in patients with essential hypertension [6]. These alterations were stratified into the following three categories according to PRAT thickness: PRAT1 (≤9.2 mm); PRAT3 (>12.9 mm); and PRAT2, with a size between the previous two values [6]. Specifically, the 24 h mean systolic blood pressure (SBP) was higher in the PRAT3 group than in the PRAT1 group (138.34 vs. 128.73 mmHg, *p* < 0.001), along with several biochemical parameters, such as triglycerides, uric acid, and homocysteine [6]. Additionally, patients with PRAT3 accumulation had significantly higher prevalences of nocturnal hypertension, morning hypertension, and a reverse dipper blood pressure profile compared with those with reduced PRAT thickness [6]. Moreover, each 1 mm increase in PRAT thickness was associated with a 0.673 mmHg increase in 24 h SBP, a 0.96 mmHg increase in morning SBP, and a 0.689 mmHg rise in nocturnal SBP [6]. Finally, conventional obesity markers, including BMI, visceral fat area, and total fat area, were not significantly associated with these alterations in the same study [6]. Therefore, assessment of PRAT thickness may help identify hypertensive patients with abnormal circadian blood pressure patterns, particularly nocturnal and morning hypertension.

Recent evidence has also highlighted the potential role of PRAT accumulation as a key factor contributing to atherosclerotic cardiovascular risk, mainly in patients with T2DM [8,35]. In this context, a positive correlation between PRAT thickness, as assessed by CT, and subclinical atherosclerosis (r = 0.401, *p* < 0.001), as measured by carotid intima-to-media thickness, was reported in a cohort of 670 participants with T2DM, supporting the potential role of PRAT thickness as a predictor of atherosclerosis in this population [8]. Another research group also demonstrated a statistically significant correlation between mean PRAT values and both blood pressure levels and carotid intima-media thickness in patients with T2DM, with a right PRAT-area thickness of 12.4 ± 4.1 mm vs. 6.5 ± 2.9 mm in controls [35].

In the same context, recent data have demonstrated that PRAT thickness is an independent predictor of 10-year ASCVD risk [1]. In addition, this association was stronger in women than in men, as reflected by the higher OR values in women (1.34 vs. 1.22; *p* < 0.001 vs. *p* = 0.009) [1]. Similarly, another recent meta-analysis revealed a positive association between PRAT expansion and established predictors of atherosclerosis, such as carotid intima-media thickness and the aortic arch calcification index (ES = 0.54, 95% CI: 0.38–0.70, I^2^ = 0.00%) [36]. Furthermore, PRAT imaging assessment may accurately reflect the dyslipidaemic metabolic profile associated with atherosclerotic risk, with PRAT volume being correlated with triglyceride (TG), total cholesterol, and low-density lipoprotein (LDL) cholesterol serum levels, along with waist circumference and BMI [5]. However, PRAT thickness increases with age, while BMI remains relatively stable after the age of 30 years in both genders [34]. These observations suggest that PRAT thickness might be a more accurate reflection of total adipose tissue accumulation than BMI. Furthermore, increased PRAT thickness does not necessarily correspond to a higher BMI, as aging is commonly associated with sarcopenia and a shift toward central fat accumulation [34]. Therefore, PRAT thickness may provide a more accurate assessment of cardiovascular risk than BMI alone [34].

PRAT expansion has also been independently associated with an increased risk of lower-extremity peripheral arterial disease (PAD) [37]. Patients with PAD have a significantly higher mean PRAT area (15.7 ± 3.7 mm) than patients without lower-extremity PAD (11.3 ± 4.4 mm) [37]. Accordingly, strategies targeting PRAT accumulation may be valuable in mitigating both the development and the progression of lower-extremity PAD.

Ethnic variation may also influence fat accumulation and PRAT-associated cardiometabolic risk, considering that Asian populations exhibit approximately 3–5% higher total body fat than White Europeans at equivalent BMI, making them more susceptible to obesity and at higher risk of CVD and/or T2DM [31]. However, these findings require validation in future studies, as most recent research has not reported participants’ ethnicity, thereby limiting the applicability of PRAT-related cardiovascular risk estimates across diverse populations.

Finally, the assessment of PRAT thickness and its association with cardiovascular risk may be influenced by methodological heterogeneity across studies. Although PRAT volume can be evaluated using various imaging methods, including CT, MRI, and ultrasonography, no universally accepted standard for its assessment has yet been established [7,31]. Variations in imaging protocols, anatomical landmarks, quantification methods, study design, and cohort characteristics, together with modality-specific limitations, such as inter-observer variability and limited ultrasound reproducibility, CT-related radiation exposure, and the higher MRI cost, contribute to substantial heterogeneity, thereby limiting the comparability, reproducibility, and interpretation of findings across studies.

Consequently, the implementation of a standardized protocol for imaging-based PRAT thickness measurement that is feasible and readily applicable in routine practice, may provide clinically meaningful insights into atherosclerotic and hypertensive risk, particularly in patients with obesity.

## 4. Molecular Mechanisms of PRAT Dysfunction in Atherosclerotic Cardiovascular Disease

Given the well-established association between obesity and ASCVD [38], extensive research has focused on elucidating the complex molecular and pathophysiological mechanisms through which excess adipose tissue contributes to the initiation, progression, and destabilisation of atherosclerotic lesions.

Epidemiological data indicate that obesity prevalence increased by 11.4 percentage points, from 30.5% in 1999–2000 to 41.9% in 2017–2020, and is projected to reach approximately 51% by 2030 [39,40]. Consistent with these trends, the 2025 World Obesity Atlas projects that, without an effective intervention, nearly 3.3 billion adults will be overweight or obese by 2035, amounting to approximately half of the global adult population [41]. Consequently, the incidence of ASCVD, the leading cause of worldwide mortality and disability, is expected to substantially increase in the coming years [41].

### 4.1. PRAT and Foam-Cell Formation

ASCVD is an umbrella term that includes ischaemic heart disease, PAD, and ischaemic stroke. Its pathogenesis is closely linked to obesity-related adipose tissue dysfunction, which is characterised by chronic low-grade inflammation and imbalanced adipokine secretion [42]. This obesity-associated pro-inflammatory visceral fat milieu promotes insulin resistance in adipocytes and impairs their capacity for efficient lipid storage, leading to systemic metabolic dysregulation [8,10]. As a consequence, excess lipids are redirected toward ectopic sites such as the liver. Therefore, impaired adipocyte storage capacity leads to lipid accumulation and increased LDL hepatic production, contributing to atherogenesis [10]. Exposure to free radicals and oxidative stress linked to obesity induces structural changes in LDL, leading to the oxidised LDL (oxLDL) synthesis, a key factor in ASCVD development and progression [43]. In recent years, oxLDL has also been increasingly recognised for its pivotal role in endothelial dysfunction and vascular inflammation associated with atherosclerosis, although the underlying mechanisms remain only partially understood [44]. However, studies have demonstrated that oxLDLs upregulate vascular adhesion molecules and monocyte chemotactic protein-1 (MCP-1) expression, promote pro-inflammatory cytokine release, stimulate the proliferation of vascular smooth muscle cells (VSMCs), and impair endothelial nitric oxide synthase (eNOS) activity [44,45].

**Figure 2:**
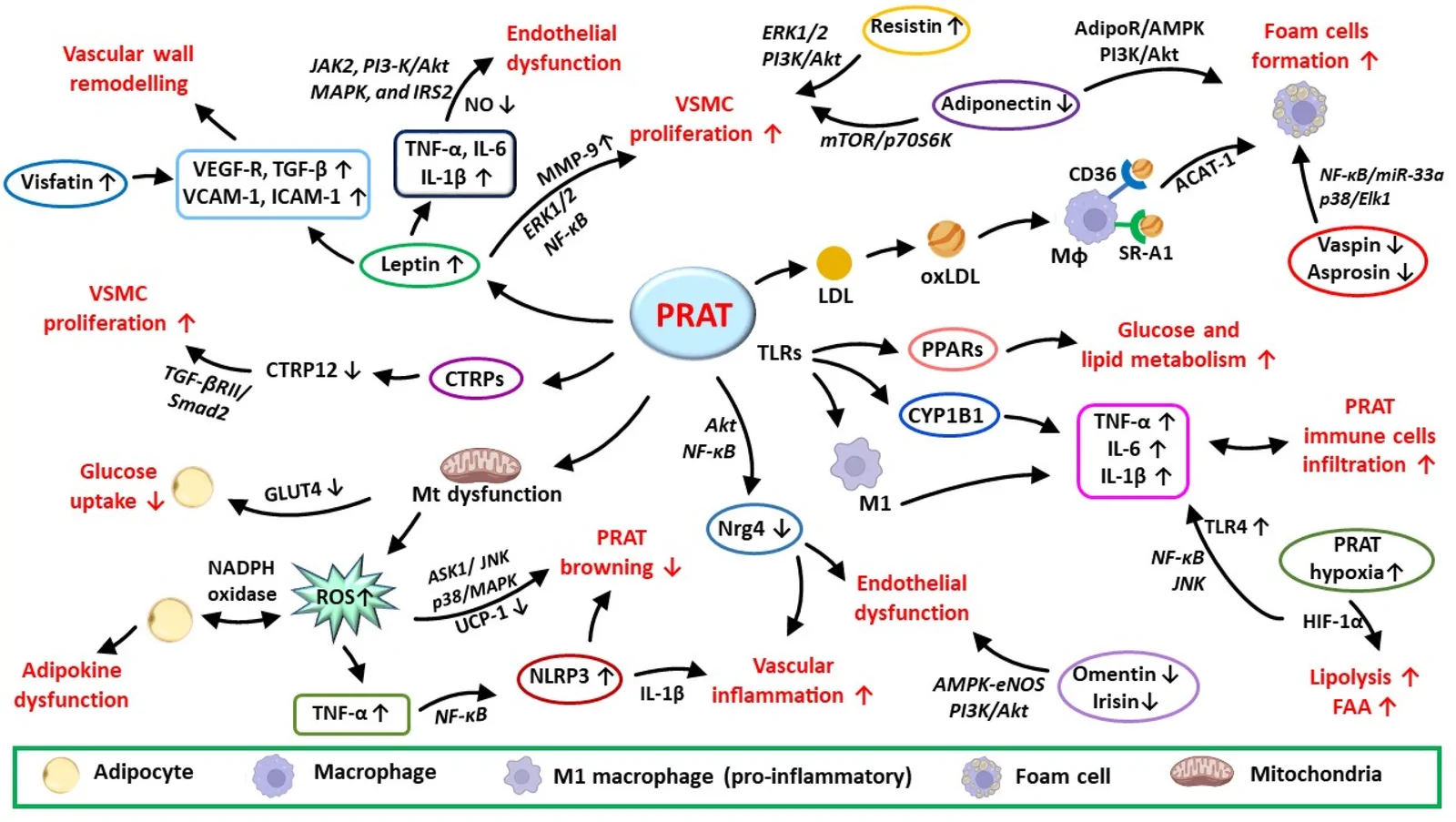
Potential mechanisms of PRAT involvement in atherosclerotic cardiovascular disease. Endothelial dysfunction is promoted by an imbalance in the secretory profile of PRAT, characterized by enhanced leptin production accompanied by reduced levels of protective adipokines, including omentin and irisin. In addition, mitochondrial dysfunction in PRAT adipocytes enhances ROS generation, thereby contributing to oxidative stress and vascular-wall inflammation. The proliferation of vascular smooth muscle cells (VSMCs) is further stimulated by increased leptin and CTRP levels, promoting vascular remodelling. Pro-inflammatory cytokines, particularly TNF-α and IL-6, exacerbate lipolysis and facilitate immune-cell infiltration into PRAT, especially by macrophages, thereby supporting local and systemic inflammation. Furthermore, increased levels of oxLDL, together with reduced PRAT-derived adiponectin, vaspin, and asprosin, promote macrophage foam-cell formation within the vascular wall, accelerating the initiation and progression of atherosclerotic plaque development. ACAT-1—acetyl-coenzyme A acetyltransferase 1; CTRPs—C1q/TNF-related proteins; CYP1B1—cytochrome P450 1B1 enzyme; GLUT4—glucose transporter type 4; HIF-1 α—hypoxia-inducible factor 1 α; ICAM-1—intracellular adhesion molecule; IL—interleukin; LDL—low-density lipoprotein cholesterol; Mφ—macrophage; MMP-9—matrix metalloproteinase-9; Mt—mitochondria; NLRP3—NOD-like receptor protein 3; NO—nitric oxide; Nrg4—neuregulin-4; PPARs—peroxisome proliferator-activated receptors; PRAT—perirenal adipose tissue; ROS—reactive oxygen species; TGF-β—transforming growth factor β; TNF-α—tumour necrosis factor; TLRs —Toll-like receptors; VCAM-1—vascular cell adhesion molecule 1; VEGF-R—vascular endothelial growth factor receptor; VSMC—vascular smooth muscle cell; ↑—increase; ↓—decrease.

Foam-cell formation, a key pathological event in atherosclerotic plaque development, is also initiated by macrophage uptake of oxLDLs, a process mediated by different receptors, such as class A1 scavenger receptors (SR-A1), lectin-like oxidised LDL receptor-1 (LOX-1), and CD36 (Figure 2) [46]. Additionally, a recent meta-analysis demonstrated that patients with ASCVD have significantly higher oxLDL concentrations than individuals without ASCVD, suggesting the potential value of oxLDL in cardiovascular risk stratification, mainly in patients with obesity [46]. These findings support the role of oxLDL as a promising biomarker for the identification of subclinical vascular damage and prediction of adverse outcomes, particularly in obesity-related conditions characterised by low-grade chronic inflammation [45].

### 4.2. PRAT Inflammation, Hypoxia, and Mitochondrial Dysfunction in ASCVD

PRAT adipocytes express Toll-like receptors (TLR2 and TLR4), linking excess adiposity to immune activation [45,47]. In obesity, TLR signalling within visceral adipose tissue promotes immune-cell recruitment and drives macrophage polarisation toward the pro-inflammatory M1 phenotype [45,47]. This activation triggers sustained secretion of pro-inflammatory cytokines and chemokines (IL-1β, IL-6, and TNF-α), establishing a self-perpetuating network of low-grade chronic inflammation of visceral fat areas, including PRAT (Figure 2) [8,9,48]. TLR-mediated responses are also modulated by nuclear receptors such as PPARs, which regulate lipid and glucose metabolism, as well as by the cytochrome P450 1B1 (CYP1B1) enzyme, which is involved in oxidative stress and inflammatory signalling. The crosstalk between adipose tissue’s TLRs, PPARs, and CYP1B1 and the vascular system plays a critical role in the development of adipose tissue dysfunction and atherosclerosis. As a result, the deciphering of these interconnected pathways is essential for the identification of novel therapeutic targets in cardiometabolic disease [10].

During obesity-associated PRAT expansion, impaired vascularisation leads to local hypoxia, which induces hypoxia-inducible factor 1 α (HIF-1α) overexpression and subsequently activates NF-κB-dependent cytokine production (e.g., IL-1β), also promoting immune-cell recruitment and shifting adipose tissue towards a pro-inflammatory state [1,13]. Concomitantly, hypoxia-driven adipocyte dysfunction increases lipolysis and free fatty acid (FAA) release, inducing endoplasmic reticulum stress and adipocyte apoptosis [1,13]. In response, macrophages surround the dying adipocytes, forming crown-like structures, and the infiltrating macrophages generate excessive reactive oxygen species (ROS) and nitric oxide (NO), further damaging the tissue, inducing fibrosis and progressive PRAT dysfunction [13]. Moreover, obesity-associated hypoxia induces TLR4 activation and downstream signalling pathways, including those of c-Jun N-terminal kinase (JNK) and NF-κB, thereby sustaining and amplifying vascular-wall inflammation [10]. Overall, PRAT hypoxia and TLR activation converge to amplify inflammatory signalling, driving chronic low-grade inflammation, metabolic dysfunction, and the progression of ASCVD in obesity.

Mitochondrial dysfunction in adipocytes also represents a key component of the visceral adipose tissue dysfunction associated with obesity. It is characterised by reduced oxidative capacity and impaired mitochondrial activity, which additionally contribute to insulin resistance and cardiorenal metabolic syndrome [10]. Mitochondrial transcription factor A (TFAM), a significant structural and functional regulator of the adipocyte mitochondrial genome, is disrupted in obesity, leading to a decreased mtDNA copy number and an altered electron transport-chain composition, resulting in ineffective respiration and metabolic uncoupling [49]. Additionally, although insulin-stimulated Akt phosphorylation is increased, the glucose uptake in adipocytes is impaired due to defective glucose transporter type 4 (GLUT4) translocation, reflecting the uncoupling of insulin signalling from the metabolic response [50]. Mitochondrial dysfunction in visceral adipocytes, including PRAT, also increases ROS production and promotes vascular inflammation, thereby contributing to atherosclerosis progression [51]. Taken together, these mitochondrial alterations reprogram the visceral adipocyte metabolism, consequently promoting insulin resistance and vascular-wall inflammation and accelerating the atherogenesis mechanism.

### 4.3. PRAT-Derived Adipokine Profile Dysfunction in ASCVD

Emerging evidence indicates that obesity-associated oxidative stress in PRAT plays a pivotal role in adipokine dysregulation, contributing to metabolic disease and ASCVD development and progression [8]. Nicotinamide adenine dinucleotide phosphate (NADPH) oxidase constitutes a major source of reactive oxygen species (ROS) in adipocytes that support the dysregulated adipokine profile in obesity [10,52]. This altered profile is characterised by increased secretion of proatherogenic adipokines (e.g., leptin and visfatin) and decreased synthesis of atheroprotective adipokines (e.g., adiponectin, omentin, and irisin) (Figure 2) [10,52].

Leptin, an adipokine whose circulating levels are markedly increased in obesity [24], has been extensively investigated for its role in the pathophysiology of ASCVD [53]. Substantial evidence shows that leptin has pleiotropic proatherogenic effects by promoting endothelial dysfunction, local inflammation, and vascular remodelling [53]. In this regard, leptin has been shown to induce macrophage recruitment and activation in the vascular wall [53]. In addition, leptin may increase intracellular cholesteryl ester accumulation, facilitating the formation of macrophage-derived foam cells within the intimal layer by upregulation of acetyl-coenzyme A acetyltransferase 1 (ACAT-1), a hallmark pathological feature of atherosclerosis [53]. These findings are supported by previous preclinical investigations that showed that leptin deficiency slows the development of atherosclerotic plaque and reduces foam-cell formation through the downregulation of CD11b, CD36, MHC class II, CD40, and SR-A expression [54].

Increased PRAT-derived leptin has also been shown to exhibit proatherogenic effects on endothelial cells through interaction with the long isoform of the leptin receptor, which activates intracellular pathways associated with local inflammatory responses in obesity [1,8]. Thereby, leptin supports the overexpression of pro-inflammatory cytokines, such as C-reactive protein (CRP), MCP-1, IL-6, and TNF-α, which increase endothelial inflammation, contributing to a proatherogenic milieu [9,53]. PRAT-derived leptin may also promote endothelial dysfunction by activation of Janus kinase 2 (JAK2), insulin receptor substrate 2 (IRS2), PI3-K/Akt, and mitogen-activated protein kinase (MAPK) pathways, which impair NO bioavailability, promote oxidative stress, and facilitate the vascular-wall inflammation and remodelling [9,53]. Moreover, leptin induces the overexpression of cell adhesion molecules, including vascular cell adhesion molecule 1 (VCAM-1) and intracellular adhesion molecule (ICAM-1), as well as angiogenic and profibrotic factors such as vascular endothelial growth factor receptor (VEGF-R) and transforming growth factor β (TGF-β), which promote leukocyte adhesion and their endothelial recruitment, support angiogenesis, and contribute to vascular fibrotic remodelling associated with atherosclerosis [53,55]. PRAT-derived leptin may also support VSMC migration and neointima formation through the overexpression of matrix metalloproteinase-9 (MMP-9) and activation of ERK1/2 and NF-κB signalling pathways [9,53,56,57].

Resistin is another PRAT-derived pro-inflammatory adipokine overexpressed in obesity, contributing to VSMC proliferation and cardiovascular dysfunction by the activation of ERK1/2, PI3K, and Akt signalling cascades [58]. In addition, visfatin, another PRAT-derived adipokine, displays its pro-atherogenic effects by stimulation of VMSC differentiation and vascular-wall inflammation [58]. Resistin also induces the expression of MMPs, VEGF, MCP-1, FGF2, and endothelial adhesion molecules (e.g., ICAM-1, VCAM-1, and E-selectin), which support leukocyte recruitment in the vascular wall and angiogenesis [59].

Ceramides, bioactive lipids accumulated in obesity, also disrupt metabolic homeostasis and may contribute to cardiovascular dysfunction through PP2A stimulation and subsequent dephosphorylation of the eNOS–Akt complex [60]. Elevated ceramide levels have been correlated with adverse cardiovascular outcomes, underscoring its potential use as therapeutic targets in ASCVD [60].

The effects of protective adipokines, such as vaspin, omentin, irisin, asprosin, and adiponectin, are significantly reduced in obesity, contributing to imbalanced adipokine signalling, which supports vascular inflammation and the development of atherosclerotic lesions. Under physiological conditions, vaspin exhibits vasculoprotective actions by preventing endothelial-cell apoptosis through activation of the PI3K/Akt signalling pathway and by inhibition of foam-cell formation within the vascular wall via NF-κB/miR-33a axis modulation [10]. Similarly, irisin and omentin improve endothelial function through the stimulation of AMPK-eNOS and phosphoinositide 3-kinase/protein kinase B (PI3K/Akt) signalling cascades, respectively [10]. Akt-mediated phosphorylation of eNOS enhances NO bioavailability, leading to vasodilation, reduced oxidative stress and inflammation, along with the preservation of endothelial homeostasis [10]. In addition, asprosin contributes to vascular homeostasis through the activation of p38/Elk1 signalling and a reduction in lipid accumulation in macrophages owing to the high expression of ABCA1 [10].

PRAT-derived adiponectin, a classical adipokine, also exhibits multiple antiatherogenic and vasculoprotective effects [8,9,26]. These actions include the attenuation of vascular inflammation by promoting M2 macrophage polarisation, inhibiting foam-cell formation, together with modulation of neoangiogenesis via AdipoR/AMPK and PI3K/Akt-dependent mechanisms and suppression of VSMC proliferation by modulation of the mTOR/p70S6K signalling axis [10]. Nevertheless, adiponectin remains one of the most controversial adipokines in the context of ASCVD, as the beneficial effects consistently demonstrated in experimental models have not been constantly noticed in clinical studies [57]. For instance, an elevated serum adiponectin level has been associated with an adverse cardiovascular outcome and increased mortality in patients with advanced chronic kidney disease or severe chronic heart failure, a phenomenon known as the “adiponectin paradox’’ [10].

Furthermore, recent experimental evidence has also highlighted the complex and potentially gender-dependent role of adiponectin in atherosclerosis [57]. Specifically, female mice with decreased adiponectin expression exhibited an increased susceptibility to atherosclerotic lesion development compared to male mice. Furthermore, according to the same study, a 50% reduction in adiponectin expression resulted in decreased plaque formation in male mice [57]. The atherosclerotic plaque reduction was also associated with decreased MCP-1 levels and colony-stimulating factor 3 (CSF3) overexpression in male mice, while the opposite pattern was observed in female mice [57]. Taken together, these findings support the multifaceted and context-dependent nature of adiponectin in ASCVD pathophysiology. The apparent duality of adiponectin-mediated effects in ASCVD development reflects the considerable complexity of the molecular crosstalk between adipose tissue and the vascular wall, which is supported by the simultaneous activity of multiple mediators, gender-related biological differences, and inter-individual variability of receptor sensitivity and of downstream signalling efficiency.

Finally, C1q/TNF-related proteins (CTRPs) constitute a distinct family of adipocyte-derived mediators with significant cardioprotective and antiatherogenic properties. Among these, CTRP9 may promote vasodilation via activation of the AdipoR/AMPK/eNOS/NO signalling axis, thereby supporting endothelial homeostasis [61]. In addition, CTRP12 shows vasculoprotective effects via TGF-βRII/Smad2 signalling cascade modulation, leading to decreased VSMC proliferation [61].

### 4.4. PRAT Brown Adipocyte Dysfunction in ASCVD

Due to its unique composition, including brown adipocytes with thermogenic function, PRAT also exerts vasculoprotective effects through the secretion of neuregulin-4 (Nrg4), which attenuates the development of atherosclerotic lesions by reducing vascular inflammation and endothelial dysfunction via Akt/NF-κB signalling-pathway modulation [10,26] (Figure 2). However, obesity-associated chronic inflammation significantly impairs the physiological function of PRAT brown adipocytes, thereby contributing to metabolic dysfunction and the progression of atherogenic dyslipidaemia [10]. This process is at least in part driven by persistent exposure to pro-inflammatory cytokines (e.g., IL-1β and TNF-α) and sustained NF-κB activation, which, together, increase metabolic inflammation and suppress the thermogenic program of brown adipocytes [62].

A central mediator of this inflammatory response is the NOD-like receptor protein 3 (NLRP3) inflammasome, a key component of the innate immune response within adipose tissue [62]. TNF-α, a pro-inflammatory cytokine overexpressed in obesity, acts as a major upstream regulator of adipocyte NLRP3 inflammasome activation, induces UCP-1 downregulation, and reduces mitochondrial respiration [62]. The NLRP3 inflammasome also reduces the thermogenic capacity of PRAT and enhances the production of inflammatory cytokines by the activation of the adipocyte TLR/NF-kB signalling pathway [62]. Nevertheless, emerging evidence demonstrates that inflammatory mediators may show context-dependent effects within PRAT. Different studies have shown that a transient increase in IL-6 expression and NF-κB activation induced by cold exposure may also promote PRAT browning through UCP-1 overexpression and activation of the GP130/STAT3 signalling pathway [63,64]. These findings suggest that IL-6 and NF-κB may also function as physiological adaptive regulators under particular metabolic conditions [62]. However, depot-specific differences have also been identified in the browning process of adipose tissue. Accordingly, browning of subcutaneous white adipose tissue is generally associated with IL-10 overexpression and enhanced adiponectin secretion, both of which have cardioprotective effects. In contrast, visceral adipose tissue, including PRAT, exhibits IL-6 and TNF-α overexpression, even during browning, a feature most likely related to its increased hypoxia and its abundant immune-cell infiltration [62].

Adipocyte browning and thermogenesis suppression are also driven by excessive ROS generation in the PRAT of patients with obesity, inducing activation of the ASK1-dependent JNK/p38 MAPK signalling pathway [62]. Experimental studies have revealed that ASK1-deficient mice display increased beige adipocyte formation, characterised by UCP-1 overexpression, decreased systemic inflammation, and improved insulin resistance, identifying ASK1 as a potential therapeutic target in obesity-associated cardiometabolic disease [62].

Taken together, obesity-associated inflammation establishes a complex molecular network that suppresses adipocyte browning and thermogenic activity in PRAT depots. Pharmacological or genetic disruption of NLRP3, TLR4/NF-κB, or ASK1 signalling pathways has been shown to restore thermogenesis, to enhance energy expenditure, to improve glucose metabolism, and to provide cardiovascular protection. Conversely, impaired thermogenic function promotes pro-inflammatory adipokine secretion, leading to endothelial dysfunction and vascular inflammation, thereby negatively affecting vascular-wall integrity.

## 5. Molecular Mechanisms of PRAT Dysfunction in Hypertension

Considering its close anatomical association with the kidneys, the PRAT depot may influence renal function, contributing to the development of hypertension, a highly prevalent disease affecting 1.39 billion people worldwide [9,65]. Consistent evidence suggests that PRAT expansion in obesity may contribute to the development of hypertension through both mechanical and neurohumoral mechanisms [5,12].

### 5.1. Mechanical and Neurogenic Regulation of PRAT in Hypertension

**Figure 3:**
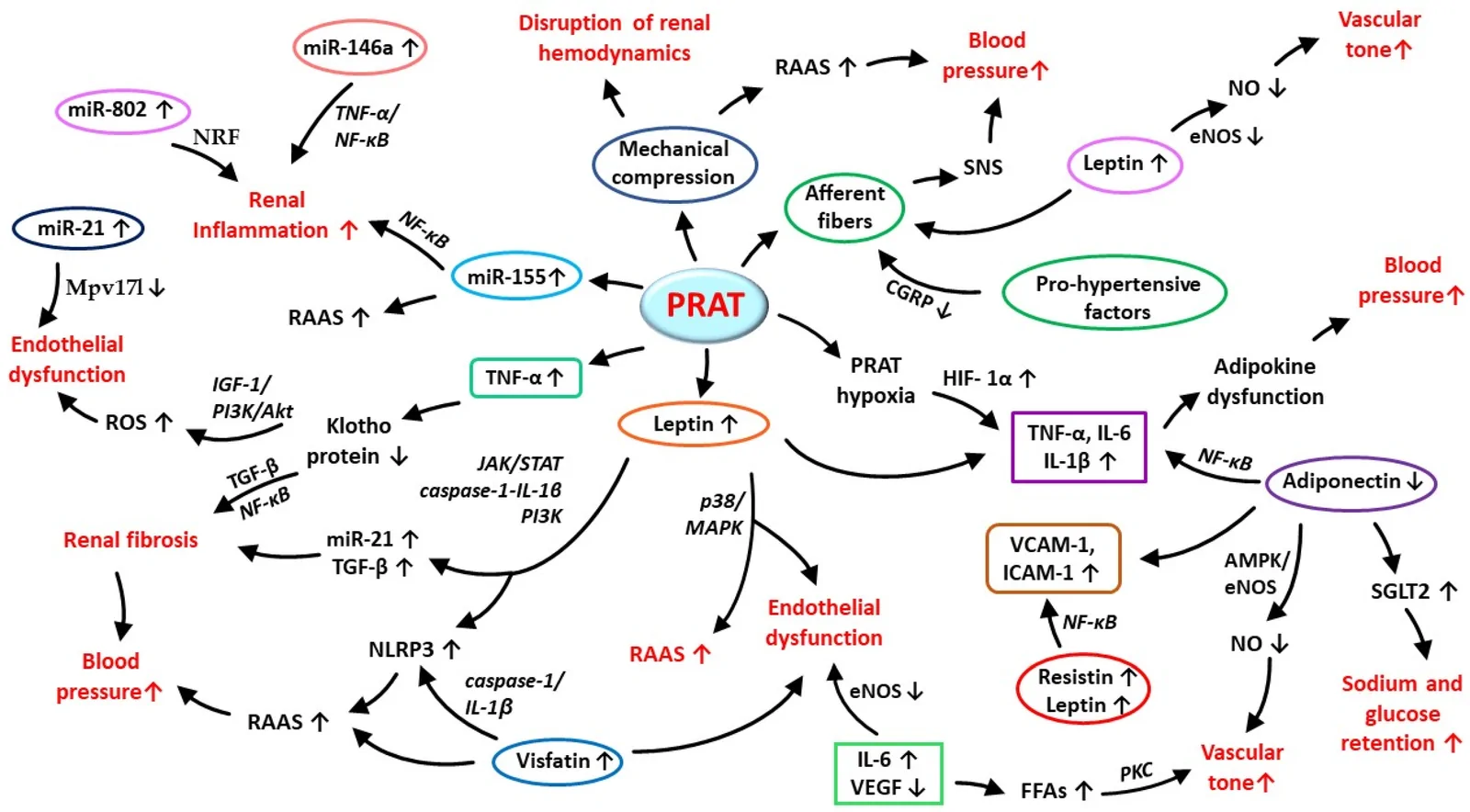
Potential mechanisms of PRAT in hypertension. Endothelial dysfunction may result from increased secretion of PRAT-derived leptin and TNF-α, as well as from the direct mechanical compression of the renal parenchyma by excessive PRAT. Leptin further contributes to vascular dysfunction by promoting vasoconstriction and activation of the renin–angiotensin–aldosterone system (RAAS), whereas activation of renal afferent sensory nerves within PRAT enhances renal sympathetic nerve activity, leading to sustained elevated blood pressure. Concurrently, reduced adiponectin secretion of PRAT promotes renal sodium and glucose reabsorption via increased sodium–glucose cotransporter 2 (SGLT2) activity. These effects are further amplified by the upregulation of miR-802, miR-146a, and miR-155, which contribute to increased vascular tone, renal fibrosis, and hypertension. CGRP—calcitonin gene-related peptide; eNOS—endothelial nitric oxide synthase; HIF-1 α—hypoxia-inducible factor 1 α; miR—microRNA; Mpv17l–mitochondrial inner membrane protein-like 17l; NLRP3—NOD-like receptor protein 3; NO—nitric oxide; NRF—NF-κB repressing factor; PRAT—perirenal adipose tissue; RAAS—renin–angiotensin–aldosterone system; ROS—reactive oxygen species; SGLT—sodium–glucose cotransporter 2; SNS—sympathetic nervous system; TGF-β—transforming growth factor β; TNF-α—tumour necrosis factor; VEGF—vascular endothelial growth factor; ↑—increase; ↓—decrease.

As PRAT volume increases, it may compress different renal structures, impairing renal perfusion, reducing the tubular flow, promoting sodium reabsorption, and consequently contributing to elevated arterial blood pressure [5,12,22,66,67]. This process is attributed to the specific location of PRAT and to the morphology of the renal fascia, a dense connective tissue that envelops the PRAT area and displays limited elasticity [68]. Beyond the direct mechanical disruption of the renal haemodynamics, chronic renal compression due to obesity-related PRAT expansion may stimulate renin release by the juxtaglomerular cells, leading to RAAS activation and, consequently, increased blood pressure (Figure 3) [12]. The classical RAAS pathway includes angiotensin-converting enzyme (ACE), angiotensin II (Ang II), and its type 1 receptor (AT1R) [69]. Under pathophysiological conditions, the activation of this axis induces vasoconstriction by acting on AT1 receptors on VSMCs and increases sodium retention in renal tubules, contributing to the development of hypertension [69].

In addition to its mechanical effects, PRAT exhibits distinctive neural features that may influence blood pressure homeostasis [4,68]. Sensory afferent fibres within this fat area communicate with the sympathetic nervous system through a feedback mechanism called the “adipose afferent reflex” [5]. Enhanced afferent signalling from the PRAT area has been associated with increased renal sympathetic nerve activity and increased blood pressure values [12]. Recent results have highlighted the presence of leptin receptors and of transient receptor potential vanilloid 1 (TRPV1) on the membranes of afferent nerve fibres in PRAT areas, which may modulate local neural signalling [68,70]. Based on these data, leptin administration into PRAT has been shown to induce the adipose afferent reflex without significantly altering the serum levels of other sympathetic activating mediators, suggesting a direct role of PRAT in blood pressure modulation [71]. Furthermore, leptin injection into visceral adipose tissue may lead to c-Fos overexpression in the paraventricular nucleus, a key site of fat-tissue afferent signalling and of sympathetic regulation, while inhibition of this nucleus blocks the sympathetic response [72]. Moreover, a study using deoxycorticosterone acetate (DOCA) salt-induced hypertension models revealed that enhanced TRPV1 activity in PRAT afferent nerves may also contribute to increased blood pressure [73]. Furthermore, blocking the activity of PRAT afferent nerves has been shown to decrease sympathetic activity and blood pressure [68]. In this regard, denervation of adipose tissue afferents using resiniferatoxin, an ultrapotent TRPV1 agonist, led to significant decreases in sympathetic nerve activity and mean blood pressure in diet-induced obese hypertensive mice, highlighting the potential value of TRPV1 as a promising therapeutic target in hypertension [72,73].

Finally, chronic pro-hypertensive factors such as high dietary salt intake, tobacco use, and sedentary lifestyle have also been suggested to support PRAT afferent nerve activation, leading to the suppression of calcitonin gene-related peptide (*CGRP*) synthesis and the limitation of its vasodilatory and antihypertensive effects [65]. Experimental data revealed that PRAT ablation restores CGRP synthesis and lowers blood pressure, suggesting the potential therapeutic role of PRAT ablation in the management of essential hypertension [65].

Afferent nerves within PRAT appear to contribute to hypertension, whereas PRAT ablation produces a sustained antihypertensive effect. However, the underlying mechanisms and the roles of specific afferent nerve subtypes remain incompletely understood. Even though PRAT ablation has been shown to attenuate sympathetic tone, it does not produce detectable changes in urinary catecholamines nor dopamine, epinephrine or norepinephrine serum levels, supporting the involvement of a neurogenic mechanism in PRAT-associated hypertension rather than systemic alteration of circulating catecholamines [65]. Additionally, selective renal deafferentation did not prevent the development of high blood pressure in hypertensive rat models, unlike PRAT ablation [66,74]. This may be attributed to the fact that kidney afferent nerves predominantly project to the T9–T13 dorsal root ganglia, whereas PRAT afferents mainly project to T13 and L1 dorsal root ganglia [68]. These differences are significant, given that L1–L2 dorsal root ganglia neurons may act as independent modulators of blood pressure [70]. Collectively, these distinct neural projections may explain the differences in neurogenic blood pressure regulation between PRAT and the kidneys, highlighting PRAT as a unique adipose depot with specific neurogenic effects that contribute to blood pressure modulation.

### 5.2. Imbalance of PRAT-Derived Adipokines and Pro-Inflammatory Cytokines in Hypertension

Dysfunctional PRAT constitutes an important source of adipokines and pro-inflammatory cytokines, possibly contributing to renal dysfunction and promoting the development of hypertension in obesity [8,9]. Expansion of PRAT without adequate compensatory vascularisation leads to local hypoxia, adipocyte death, and the emergence of a local pro-inflammatory microenvironment. PRAT hypoxia promotes HIF-1 overexpression, along with the activation of multiple pro-inflammatory signalling axes [4,13,68]. In response to hypoxia and the associated inflammatory state, PRAT production of leptin, visfatin, and resistin increases, along with reduced adiponectin expression [4,75]. Persistent hypertension is also associated with an imbalance between endothelium-derived vasodilatory (e.g., NO) and vasoconstrictive (e.g., endothelin, angiotensin II, and uridine adenosine tetraphosphate) factors due to enhanced vascular endothelial turnover in small blood vessels [76]. Furthermore, an indicator of endothelial dysfunction and systemic microvascular injury is albuminuria, which is frequently detected in prehypertensive patients and is associated with an increased risk a progressive decline in renal function [22]. In this context, inflammation-induced glomerular hyperfiltration may be considered the mechanistic link between microalbuminuria and hypertension.

PRAT-derived leptin may also act as a vasoactive factor, promoting endothelial dysfunction; sodium retention; and, therefore, the development of hypertension via activation of the p38/MAPK signalling pathway and RAAS modulation (Figure 3) [5,9,68]. Additionally, leptin overexpression in PRAT depots may contribute to endothelial dysfunction via eNOS inhibition, increased oxidative stress, pro-inflammatory factors (e.g., TNF-α and IL-6), and the overexpression of adhesion molecules [9,75]. Moreover, PRAT-derived leptin may activate JAK/STAT, caspase-1-IL-1β, and the PI3K axis, resulting in increased miR-21 and TGF-β expression, the activation of the NLRP3 inflammasome and mesangial cells, and type I collagen synthesis, leading to renal fibrosis and glomerulosclerosis, contributing to sodium retention, RAAS activation, and hypertension [4]. Renal fibrosis induces local hypoxia, which, in turn, triggers endothelial dysfunction, oxidative stress, and inflammation, accompanied by decreased NO synthesis, ultimately leading to increased vascular tone and hypertension [4]. In summary, PRAT-derived leptin dysregulation contributes to a pro-oxidative and pro-inflammatory vascular milieu that promotes the development of hypertension in obesity.

The effects of leptin are further supported by other PRAT-derived adipokines, such as visfatin and resistin. PRAT-derived visfatin overexpression in hypertensive experimental models of obesity has been associated with endothelial dysfunction, considering that visfatin stimulates NADPH oxidase activity in endothelial cells, leading to increased ROS production [77]. Excessive ROS production, induced by visfatin, also promotes NLRP3 inflammasome activation in podocytes, leading to their injury, in association with renal fibrosis and hypertension through NLRP3–caspase-1–IL-1β axis activation [4].

Increased ROS synthesis and endothelial dysfunction are also supported by resistin, another pro-inflammatory adipokine that induces MCP-1, ICAM-1, and VCAM-1 overexpression via NF-κB signalling-pathway activation [78].

Several studies have reported a link between decreased adiponectin synthesis and hypertension development across different experimental models [68,78]. Adiponectin exerts vasculoprotective effects by stimulation of eNOS activity, downregulation of the expression of adhesion molecules, suppression of inflammatory responses, and inhibition of the NF-κB pathway [75]. In this context, AdipoRon, an agonist of AdipoR1/2, has been demonstrated to counteract the metabolic effects of TNF-α and to contribute to endothelial homeostasis by modulating lipid metabolism in human umbilical-vein endothelial cells (HUVECs) [79]. PRAT-derived adiponectin may also show inhibitory effects of SGLT2 expressed in renal tubular epithelial cells, leading to impaired sodium and glucose co-transport in the renal parenchyma, as observed in murine models following administration of a high-salt diet for 24 weeks, highlighting its role in blood pressure homeostasis [80]. Decreased adiponectin expression also contributes to reductions in the activity of eNOS and of the synthesis of NO, a master modulator of vascular relaxation [4]. This effect is partly attributed to the inhibition of AMPK signalling in renal tubular cells, promoting ROS synthesis and oxidative stress [4]. Moreover, obesity-associated adiponectin deficiency induces MCP-1 upregulation via MAPK pathway modulation, leading to renal fibrosis, M2 phenotype macrophage activation, and increased TGF-β synthesis [4].

Pro-inflammatory cytokines released by obesity-related expanded PRAT also contribute to the development of hypertension by promoting renovascular endothelial dysfunction. Among these pro-inflammatory cytokines, TNF-α and IL-6 are key PRAT-derived cytokines involved in the pathogenesis of hypertension [5]. In this process, an increased PRAT-derived TNF-α synthesis reduces Klotho protein expression in renal epithelial cells via NF-κB activation, leading to enhanced TGF-β secretion and renal fibrosis [4]. In addition, Klotho protein deficiency promotes ROS generation and oxidative stress through activation of the IGF-1/PI3K/Akt signalling axis, which enhances NADPH oxidase activity and contributes to endothelial dysfunction and hypertension [4]. TNF-α released by PRAT also reduces NO synthesis in renal endothelial cells, thereby promoting endothelial dysfunction and consequently impairing the glomerular filtration rate [68]. NO production is also modulated by PRAT-derived IL-6 overexpression, which reduces eNOS activity and increases MCP-1 production via modulation of the JAK/STAT signalling pathway [4]. Additionally, MCP-1 promotes the activation of pro-inflammatory signalling cascades in the renal parenchyma, enhancing ROS production and profibrotic factors synthesis, which lead to increased renal inflammation and fibrosis, contributing to hypertension [9].

VEGF released by PRAT is a key regulator of renal function that supports renal endothelial cell growth and proliferation and contributes to endothelium-dependent vasodilation by inducing eNOS overexpression [81]. Altered VEGF signalling leads to endothelial dysfunction due to decreased synthesis of NO, a key vasodilatory factor [4]. VEGF overexpression is also promoted by increased FFA production, which is associated with PRAT expansion [7]. Elevated FFA synthesis induces endothelial dysfunction via increased oxidative stress and impaired NO signalling via PKC activation, leading to increased vascular tone [4]. In addition, FFA metabolites—mainly diacylglycerol and ceramides—have lipotoxic effects on renal tissue, inducing renal chronic inflammation and insulin resistance [7,68,82].

### 5.3. PRAT-Derived Extracellular Vesicles in Hypertension

More recently, exosomes have emerged as a novel research focus with potential relevance for the development of hypertension in obesity. Exosomes are a subtype of small extracellular vesicles, carrying a diverse cargo of bioactive molecules, including lipids (FFAs, ceramides, and cholesterol), DNA (both mitochondrial and genomic), and various RNA species [4,67,83]. Among the molecular components of exosomes, microRNAs (miRNAs) have attracted considerable attention in recent years due to their potential role in blood pressure modulation. In this regard, dysregulation of PRAT-derived miRNA secretion in obesity has been associated with insulin resistance, renal fibrosis, and increased vascular tone, all of which contribute to the development of hypertension [4]. Thus, PRAT-derived miR-155 overexpression has been demonstrated to contribute to RAAS activation (Figure 3). miR-155 also promotes renal inflammation, microalbuminuria, and the development of hypertension via modulation of the NF-κB signalling pathway, leading to overexpression of TNF-α and IL-6, along with downregulation of SHIP1/INPP5D, a significant anti-inflammatory mediator [4,84]. The pro-inflammatory TNF-α/TGF-β1/NF-κB cascade is also supported by increased miR-146a expression in PRAT, promoting renal fibrosis, sodium retention, and increased blood pressure [4].

Renal dysfunction and increased vascular tone may also be mediated by miR-802 overexpression, which contributes to renal inflammation by inhibiting NF-κB repressing factor (NRF) activity [85] (Figure 3). Furthermore, increased PRAT-derived miR-21 expression has been related to microalbuminuria and endothelial dysfunction through mitochondrial inner-membrane protein-like 17l (Mpv17l) downregulation, a process that contributes to increased ROS generation, decreased NO synthesis, and increased vascular tone [4].

Finally, a recent study demonstrated that increased PRAT-derived miR-24-3p, a regulator of CYP11B2, correlates with serum aldosterone levels, PRAT volume, and BMI and therefore may contribute to increased cardiovascular risk in patients with primary aldosteronism [67]. In the same context, another prospective UK Biobank study that explored the association between PRAT and hypertension showed that PRAT thickness was associated with higher risk of essential hypertension in idiopathic hyperaldosteronism and low-renin essential hypertension [83].

Collectively, these findings highlight the complexity of the mechanisms used by PRAT-derived molecules in their contribution to renal physiology and vascular-tone homeostasis. Nevertheless, their precise roles in PRAT involvement in hypertension remain to be fully elucidated in future clinical studies, especially given that the complex in vivo hormonal and cellular environment may substantially influence the experimental outcome.

## 6. PRAT-Based Therapeutic Strategies in Cardiovascular Diseases

### 6.1. Lifestyle Interventions

The current management of obesity involves a multidisciplinary approach that includes dietary changes, physical activity, pharmacological therapy, and surgical interventions, all of which may have a positive impact on CVD prevention. Consistent evidence shows that increased physical activity, alongside the adoption of a diet rich in vegetables, fruits, and fish-derived oils, reduces PRAT volume and improves metabolic status [5]. Equally, plant-derived/herbal products may represent a therapeutic option for the modulation of adipocyte activity and CVD prevention. Reductions in the size and volume of PRAT adipocytes have been observed after administration of Kurozu concentrated liquid [86], green tea extract [87], or *C. tumida* peels [88], all of which are substances with a positive impact on the obesity–PRAT–CVD axis.

Focused ultrasound (high-intensity focused ultrasound) is another emerging non-invasive therapy currently being investigated for PRAT thickness reduction. It induces adipocyte degeneration via localized heating following targeted ultrasound delivery, leading to an increased local temperature of up to 50–55 °C (13–18 °C above the baseline) for a short period of time [5]. Preliminary data from a multicentre, double-blind clinical trial (NCT06283758) suggest that this method is safe, being associated with a significant blood pressure reduction in essential hypertension due to a decrease in PRAT volume [5]. Based on these positive results, ultrasound therapy targeting PRAT represents a potential future therapeutic strategy for blood pressure modulation in patients with obesity.

### 6.2. PRAT-Derived Adipokine and Cytokine Modulation

Modulating adipokine and pro-inflammatory cytokine secretion may represent another therapeutic approach for patients with obesity. Statins increase adiponectin levels and downregulate leptin expression, leading to a lower cardiovascular risk profile via several effects, such as improvement of endothelial function, decreased VSMC proliferation, modulation of the vascular inflammatory pathways, and decreased albuminuria [89,90]. Consistent with these findings, increased adiponectin serum levels were registered after six months of pravastatin therapy in patients with obesity and coronary artery disease [91]. Although no data linking statin treatment to modulation of PRAT dysfunction are currently available, these observations suggest that statins may have a positive impact on PRAT physiology by modulating its adipokines and pro-inflammatory factor profile [13,92].

Activation of the PRAT NLRP3 inflammasome has been associated with the progression of cardiorenal dysfunction via multiple mechanisms, including IL-1β overexpression, excessive ROS generation, and lipid accumulation in podocytes via IL-1β/ROS/NF-κB p65 signalling axis modulation [15]. MCC950, an inhibitor of the NLRP3 inflammasome, has been shown to attenuate albuminuria and to improve renal function in experimental models [15]. Therefore, it may be considered a potential target in a future therapeutic approach designed to prevent adipose tissue inflammation mediated by NLRP3 inflammasome, leading to reduced cardiovascular risk.

Despite these favourable results in preclinical studies and in phase Ib clinical trials in patients with rheumatoid arthritis, MCC950-based therapy was not developed due to its high hepatotoxicity [93]. However, Nodthera has developed NT-0796, a new oral CNS-penetrant NLRP3 inflammasome inhibitor currently being evaluated for the management of obesity-associated cardiovascular risk [93]. Moreover, the association of NT-0796 with semaglutide has been demonstrated to reduce body weight, leptin serum levels, and markers of vascular and hepatic inflammation more effectively than monotherapy with NT-0796 or semaglutide in an experimental model [94]. Additionally, compound 32, a novel non-tricyclic NLRP3 inflammasome inhibitor with a positive pharmacological profile, represents another potential therapeutic agent for obesity-associated cardiovascular risk, considering its contribution to reductions in PRAT-derived inflammation [93]. However, future studies are required to establish its long-term safety and its cardiovascular benefits.

PRAT inflammation may also be modulated by metformin and pioglitazone, two oral antidiabetic agents [95]. In this context, a reduction in PRAT inflammation was observed after two weeks of administration of these agents in a prediabetic murine model [95].

Modulation of PRAT inflammation and leptin serum levels was also registered following the administration of SGLT2 inhibitors in experimental models [96] via AMPK/Sirt1 pathway activation [13]. Therefore, the administration of SGLT2 inhibitors may be another therapeutic strategy aimed at reducing major adverse cardiovascular events [97,98,99]. In line with these data, the EMPA-REG OUTCOME trial showed a 38% reduction in cardiovascular mortality and a 35% decrease in hospitalizations for heart failure following treatment with empagliflozin, an SGLT2 inhibitor that modulates renal glucose reabsorption in patients with T2DM [100]. These preliminary results are supported by other studies investigating the impact of the administration of SGLT2 inhibitors, such as canagliflozin in the CANVAS Program and dapagliflozin in the DECLARE–TIMI 58 trial [101]. Similarly, a decrease in PRAT mass was observed in a phase IV trial (EudraCT: 2019-000979-16-Omendapa trial) in patients with obesity and T2DM following dapagliflozin and metformin administration, accompanied by decreased CRP and leptin serum levels [102]. However, the mechanisms underlying SGLT2 inhibitor-associated cardiovascular protection remain only partially deciphered. Therefore, further investigations are needed to elucidate the underlying mechanisms and to establish the optimal doses to prevent adverse events associated with SGLT2 inhibitors, such as genital mycotic infections, diabetic ketoacidosis, or Fournier gangrene [4].

### 6.3. Other Pharmacological Agents Targeting PRAT in CVD

GLP-1 RAs such as liraglutide and semaglutide are among the most successful clinical examples of therapeutic agents targeting adipose tissue activity in CVD [16]. Thus, liraglutide was considered a suitable treatment for the prevention of cardiovascular events and mortality in a large cohort of T2DM patients with or without a history of NYHA functional class I–III heart failure in the LEADER trial [103]. A decrease in cardiovascular risk was also observed in the SUSTAIN-6 clinical trial, which was conducted in patients with or without microvascular disease who received a weekly dose of semaglutide [104]. Overall, these studies provide evidence that GLP-1 RA administration may exert cardioprotective effects in obese and T2DM patients. However, GLP-1 RA use may be accompanied by adverse effects, such as gastrointestinal symptoms, pancreatitis or increased thyroid cancer risk [4,105]. Therefore, further studies are needed to elucidate the mechanisms underlying their cardioprotective effects, to establish their optimal dose, and to better understand the individual differences in the therapeutic response.

Another potential therapeutic approach to prevent ASCVD is PPARγ activation in the PRAT depot. In this regard, pioglitazone, a PPARγ agonist, has been shown to decrease vascular-wall inflammation via downregulation of the expression of the endothelial adhesion molecule and inhibition of macrophage infiltration into atherosclerotic plaques [14]. However, PPARγ agonists have also shown different cardiovascular outcomes. Although they exert anti-inflammatory and anti-atherosclerotic effects, they may also promote sodium and fluid retention, thereby precipitating or worsening heart failure [4,10,14]. These findings underscore the importance of developing next-generation PPARγ modulators that preserve the beneficial metabolic and anti-inflammatory effects of PPARγ activation while minimizing their adverse effects [10,14].

Niacin has also been proposed as a potential therapeutic agent capable of decreasing systemic inflammation and promoting adiponectin overexpression, both of which contribute to the prevention of cardiovascular events [10]. Moreover, niacin may modulate lipid metabolism and reduce adipocyte lipolysis via hydroxycarboxylic acid receptor 2 (HCA2) activation [10]. Although several studies have registered reductions in myocardial infarction rates and long-term mortality, other studies have failed to confirm these positive effects of niacin administration in association with a statin [10]. In the same context, a large clinical trial (HPS2-THRIVE) involving 25,673 patients at high risk of vascular disease demonstrated that niacin–laropiprant administration for an average of 3.9 years was associated with impaired glycaemic control and increased rates of infection, bleeding, and gastrointestinal symptoms, without providing any significant reduction in cardiovascular risk [106]. Despite exhibiting limited clinical benefits in patients with CVD, niacin continues to be considered a potential therapeutic option, mainly in patients with low high-density lipoprotein (HDL) cholesterol and elevated TG and LDL cholesterol levels and who display an adequate response to first-line lipid-lowering therapies [10].

Based on the recognised relationship between PRAT, RAAS, and CVD, RAAS inhibitors have been proposed as a therapeutic tool for the prevention of cardiovascular events and mortality in patients with obesity [10]. Beyond their recognised blood pressure-modulating effect, RAAS inhibitors have been associated with a positive effect on atherosclerosis development in a murine model [107] and with reduced cardiovascular events in the HOPE and EUROPA clinical trials [108]. Their anti-atheromatous effect is provided by a decrease in oxidative stress, improving endothelial homeostasis and reducing the synthesis of pro-inflammatory cytokines in adipose tissue [10]. However, the molecular events involved in the positive cardiovascular effects of RAAS inhibitors, by targeting dysfunctional adipose tissue depots in obesity, remain incompletely understood. Their elucidation may open new pathways for a personalised therapy in selected patients.

### 6.4. Modulation of Brown Adipocyte Activity in PRAT

Given its unique morphological structure, the activation of brown adipocytes of the PRAT depot may be another promising target for the management of metabolic complications in obesity. In this regard, a study performed in young adults with obesity showed that exposure to two hours of cold stimulation was associated with a higher adiponectin-to-leptin ratio, together with reduced triglycerides and increased HDL cholesterol serum levels [109]. These results suggest that brown adipocyte activation may display protective effects against cardiometabolic disturbances. The activation of brown adipocytes in visceral fat, including PRAT, may also be supported by the administration of the β3-adrenergic receptor agonist [9]. Although previous studies have not consistently confirmed a positive effect of β3-adrenergic receptor agonists in brown adipocyte activation, more recent data suggest that mirabegron, a selective β3-adrenergic receptor agonist primarily used to relax the bladder smooth muscle, may activate these adipocytes and may improve insulin resistance [110,111,112]. These findings support the potential therapeutic value of β3-adrenergic receptor agonists in the modulation of dysfunctional PRAT activity, leading to decreased obesity-related cardiovascular risk [62]. Despite its favourable metabolic impact, high doses of mirabegron (i.e., 200 mg) have been associated with increased heart rate, arrhythmias, and hypertension, a phenomenon known as the “mirabegron paradox’’ [62,113,114]. The “mirabegron paradox’’ is attributed to the activation of the β1- and β2-adrenergic receptors, highlighting the need for the development of more selective β3-adrenergic receptor agonists able to dissociate PRAT thermogenic activation from adverse cardiovascular effects.

### 6.5. Surgical Strategies Targeting PRAT in CVD

Finally, bariatric surgery has been shown to be associated with a reduction in PRAT thickness, which, in turn, contributes to decreased blood pressure in patients with morbid obesity [11]. In addition, sleeve gastrectomy has been linked to a reduced need for antihypertensive agents, according to the same study [11]. In the same context, a very recent study demonstrated that sleeve gastrectomy and decreased PRAT mass are associated with improvements in the lipid profile, anthropometric parameters, creatinine serum levels, and blood pressure control [115] (Table 1). Thus, bariatric surgery may lead to significant reductions in cardiovascular risk and may represent a potential therapeutic strategy, particularly in patients with morbid obesity [11,116].

Overall, PRAT biology in CVD is an ongoing area of research. However, the translation of preclinical findings into clinical practice faces significant challenges, including the safety of the therapeutic agents, their ability to effectively reduce cardiovascular risk, and heterogeneous patient responses in the context of molecular complexity associated with obesity.

## 7. Future Directions of PRAT-Based Therapy in Cardiovascular Diseases

New integrated therapeutic options are currently being considered to prevent CVD in patients with obesity by targeting the PRAT depot. Recent advances in this domain have led to the identification of several promising directions for future research, including metabolic interventions, emerging cellular therapies, modulation of the gut microbiota, and innovative monitoring technologies such as artificial intelligence-assisted analysis of CT and MRI images.

**Figure 4:**
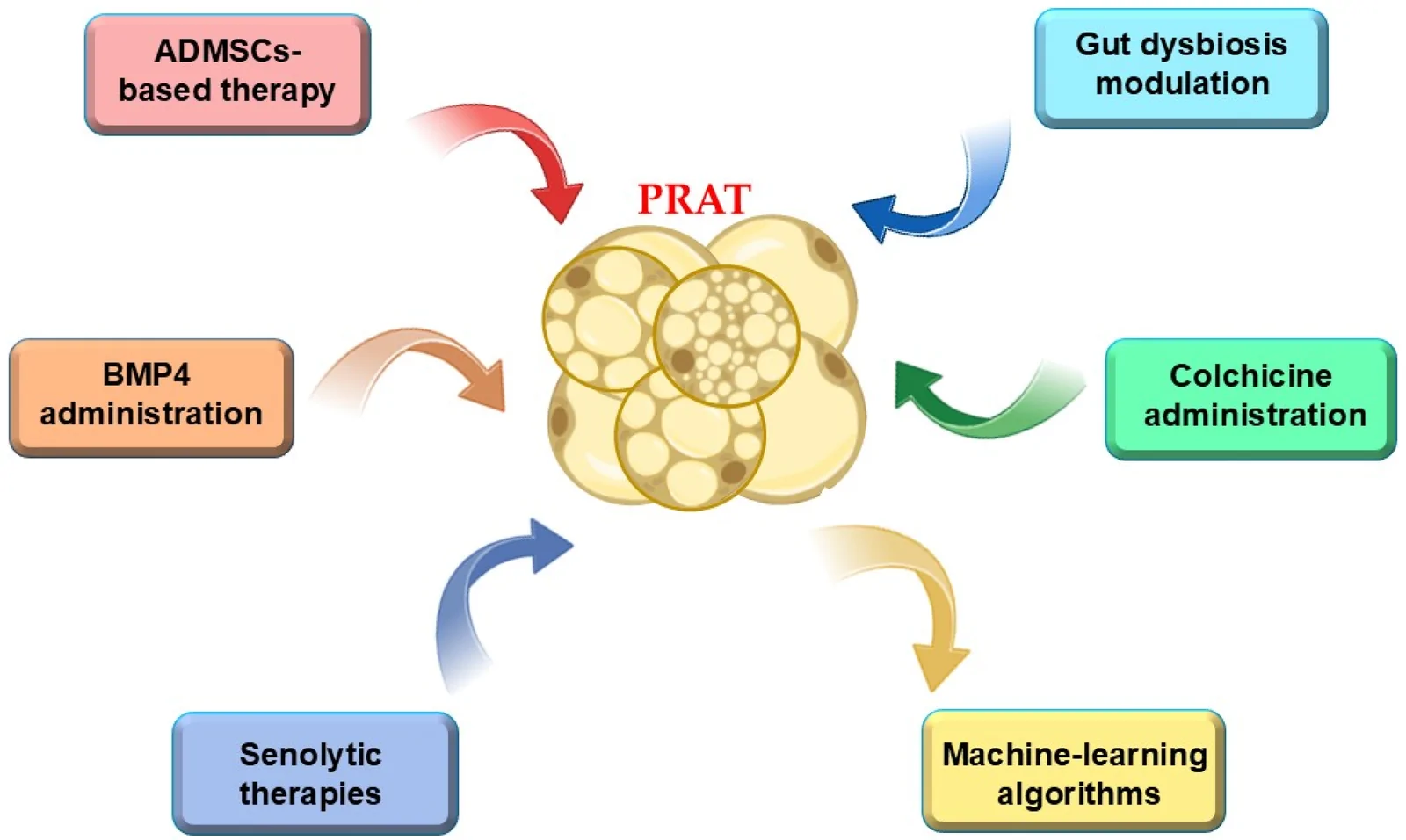
Potential PRAT-based therapies in cardiovascular diseases. Colchicine and BMP4 administration, together with modulation of the gut microbiota, adipose-derived mesenchymal stem-cell (ADMSC)-based approaches, and senolytic therapies, is an emerging therapeutic strategy for cardiovascular disease prevention in obese patients through targeted modulation of PRAT. Moreover, advanced image analysis using machine learning algorithms may further improve cardiovascular risk stratification in these patients. ADMSC–adipose-derived mesenchymal stem cell; BMP4–bone morphogenetic protein 4.

BMP4 may represent a novel therapeutic target for the regulation of the activity of visceral adipose tissue, including PRAT, due to its significant role in adipocyte differentiation and metabolic homeostasis [10] (Figure 4). Decreased BMP4 expression has been associated with downregulation of brown adipocyte-specific genes, endothelial dysfunction, and IL-1β overexpression, contributing to CVD development [18]. Furthermore, BMP4 regulates mitochondrial activity and lipid synthesis in adipocytes [18]. Its expression is linked to the maintenance of energy homeostasis and decreased fat tissue mass [18]. Additionally, exogenous BMP4 administration in experimental models has been demonstrated to attenuate hepatic steatosis and to reduce serum TG serum levels and body weight [117]. However, the positive effects of BMP4 are limited by the activity of Noggin and Gremlin-1, its endogenous antagonists, which are strongly overexpressed in obesity and contribute to BMP4 resistance [118]. Thus, given the natural barriers to direct BMP4 administration, future studies should aim to develop tissue-selective therapeutic strategies and to target BMP4 antagonists in order to overcome BMP4 resistance in adipose tissue.

Obesity is associated with an increased number of senescent cells, which can further enhance the synthesis of pro-inflammatory factors, contributing to insulin resistance, metabolic dysfunction, and CVD [10]. Consequently, current strategies aim at reducing the accumulation of senescent cells in fat tissue via senolytic therapies or limiting senescence-induced mitochondrial dysfunction have shown positive effects on metabolic parameters and adipocyte activity in experimental models [17], highlighting their potential as new therapeutic tools for CVD prevention in obesity.

Moreover, the genetic engineering strategies designed to improve ADMSC survival and their therapeutic efficacy may offer novel treatment opportunities in obesity [10]. Due to their ability to enhance endothelial functions, to decrease the vascular inflammation, to reduce oxidative stress, and to prevent vascular and renal remodelling, ADMSCs may also represent a promising therapeutic strategy for the limitation of cardiovascular risk in obese patients [10,119]. Moreover, ADMSCs may exert modulatory effects on RAAS dysregulation by modulating Angiotensin II/AT1 receptor signalling pathway and potentially promoting the counter-regulatory ACE2/Ang-(1–7)/Mas receptor axis. These mechanisms may contribute to the attenuation of vascular dysfunction and renal remodelling associated with obesity-related hypertension [120,121,122]. However, the clinical application of ADMSCs is still limited by different challenges, including poor post-transplantation cell survival and the lack of standardized protocols for their isolation and growth [119].

PRAT dysfunction may be prevented by colchicine, a well-established anti-gout agent that has been proposed as a potential modulator of CVD and metabolic diseases [10] (Figure 4). It has been shown to decrease adipocyte lipolysis in patients with obesity, leading to lower TG levels and a reduced incidence of cardiovascular events in ASCVD patients [123]. In addition, colchicine displays potent anti-inflammatory effects by decreasing NLRP3 inflammasome activation [123]. However, its positive outcomes appear to be driven primarily by systemic anti-inflammatory actions rather than a direct modulation of adipocyte activity. As a result, colchicine’s role in the modulation of adipose tissue metabolism and local inflammation remains to be fully elucidated.

Current evidence supports a correlation between gut dysbiosis and obesity [19,124]. In this regard, dysbiosis has been shown to be associated with adipose tissue expansion and inflammation by regulating the expression of genes linked to fat accumulation and restriction of FFA absorption [19,125]. Moreover, the modulation of gut dysbiosis using bioactive compounds and microbiota-derived metabolites such as bifidus factors, anthocyanins, short-chain fatty acids (SCFAs), vanillic acid, and KetoA is currently being investigated for potential beneficial effects in the obesity—dysbiosis-related complication axis [19]. Although not yet tested for PRAT in a large clinical cohort, a very recent experimental study revealed an improvement in lipid metabolism parameters and SCFA levels, along with a decrease in PRAT mass, following the administration of Epigallocatechin-3-Gallate-Quercetin-Rutin (EQR), a mixture of flavonoids, by modulation of gut dysbiosis [20]. According to the same study, a decrease in PRAT adipocyte size was associated with the downregulation of PPAR-γ and fatty acid transport protein 1 (FATP-1) expression, resulting in inhibited lipid synthesis and peroxisome proliferator-activated receptor gamma coactivator 1-α (PGC-1α) overexpression, which, in turn, promoted UCP-1 expression in PRAT adipocytes [20].

Advanced imaging techniques, such as CT and MRI, now allow for the precise assessment of the adipose tissue distribution, including of the PRAT depot. Recent advances in deep learning and MRI have also enabled automated assessment of renal tissue and PRAT thickness and the derivation of radiomic biomarkers for prediction of the pathological grade of clear-cell renal cell carcinoma [21]. Regarding cardiovascular risk, machine learning algorithms that integrate clinical variables, epicardial fat-mass assessment, and coronary artery calcium have demonstrated superior predictive performance compared with traditional risk calculators [21]. However, according to our knowledge, the prognostic role of PRAT in CVD has not been yet explored using machine learning approaches. Nevertheless, integrating both clinical and PRAT features of patients with obesity into predictive models for major adverse cardiovascular events could allow for earlier diagnosis and improved CVD risk stratification.

## 8. Conclusions

PRAT represents a unique adipose tissue depot due to the remarkable plasticity of its adipocyte population, involved in maintaining vascular homeostasis. PRAT dysfunction in obesity is characterised by enhanced local and systemic inflammation, dysregulated adipokine secretion, metabolic alterations, hypoxia, and increased oxidative stress, all of which contribute to vascular injury, atherosclerosis, and increased vascular tone.

Despite extensive research in recent decades, the underlying molecular mechanisms linking obesity-associated PRAT dysfunction to CVD development remain incompletely understood. Given the molecular and metabolic complexity of obesity, further studies are required to better characterise the interaction between PRAT adipocytes and vascular-wall morphophysiology and to support the translation of preclinical findings into clinical practice. In addition, the contributions of inter-individual PRAT morphological heterogeneity to metabolic phenotypes and cardiovascular risks are still poorly understood, underscoring the need for integrated approaches to elucidate its biological determinants and to improve the prediction of individual metabolic impact and cardiovascular risk profiles.

Although PRAT has emerged as a potential contributor to cardiovascular disease, current clinical evidence is predominantly observational and does not establish a direct causal relationship with atherosclerosis or hypertension. Consequently, longitudinal and interventional studies are needed to clarify whether PRAT is an independent determinant of cardiovascular disease or primarily a marker of systemic visceral adiposity. Besides establishing its causal impact, future research should determine whether targeted modulation of PRAT can provide any cardiovascular benefits beyond those achieved through generalized visceral adipose tissue reduction.

A deeper understanding of the full spectrum of metabolic and inflammatory disturbances associated with PRAT may provide new perspectives with respect to the refinement of personalised strategies for cardiovascular risk stratification, early diagnosis, and targeted therapies in patients with obesity.

## Figures and Tables

**Table 1:** Potential PRAT therapeutic approaches in cardiovascular diseases.

Therapeutic Approach	Intervention	Study Design	Study Population/Model	Effects	References
Lifestyle modulation	Herbally derived products	Murine model	Male C57BL/6J-*Lepob/ob* mice Four-week-old male SD rats C57BL/6J male mice	PRAT adipocyte size ↓ Lipid metabolism parameters ↓ PRAT area mass ↓	[86,87,88]
Non-invasive techniques	Focused ultrasound	Clinical trial	30 patients with essential hypertension	Adipocyte degeneration ↑ Blood pressure ↓	[5]
Statins	Pravastatin	Observational pilot study	115 patients with coronary artery disease	Adiponectin ↑ HDL cholesterol ↑ Total and LDL cholesterol ↓	[91]
NLRP3 inflammasome inhibitors	NT-0796 and semaglutide	Experimental model	hCES1 (human carboxylesterase 1) mice	Body weight ↓ Leptin ↓ sVCAM-1 ↓ Insulin resistance ↓ Liver IL-1α ↓	[94]
	Compound 32	Experimental model	C57BL/6 female mice with acute peritonitis	IL1-β ↓ Lower hepatotoxicity than MCC950	[93]
Antidiabetic agents	Metformin and pioglitazone	Experimental model	Male Sprague–Dawley rats	Glomerular filtration ↑ IL-1β ↓, TGF-β1 ↓, and VEGF ↓ Oxidative stress ↓ eNOS activity ↑	[95]
SGLT2 inhibitors	Empagliflozin	Clinical trial	7020 patients with T2DM and ASCVD	Cardiovascular risk ↓ Established ASCVD	[100]
	Canagliflozin	Clinical trial	10,142 patients with T2DM and high cardiovascular risk	Cardiovascular risk ↓ Cardiovascular events ↓	[98]
	Dapagliflozin	Clinical trial	17,160 patients with T2DM	Cardiovascular death ↓ Hospitalization for HF ↓	[99]
	Dapagliflozin and metformin	Clinical trial	29 patients with obesity and T2DM	PRAT mass ↓ CRP ↓ Leptin ↓	[102]
GLP-1 RAs	Liraglutide	Clinical trial	9340 patients with T2DM with NYHA functional class I-III HF	Risk of hospitalization ↓ Cardiovascular events ↓	[103]
	Semaglutide	Clinical trial	3297 patients with and without microvascular disease	Cardiovascular risk ↓	[104]
PPARγ agonist	Pioglitazone	Experimental model	Obese ob/ob mice and control C57BL/6 mice	Arterial stiffening ↓ Vascular-wall inflammation ↓ Macrophage infiltration of the atherosclerotic plaques ↓	[14]
Niacin	Niacin and laropiprant	Clinical trial	25,673 patients with high risk of vascular disease	No significant reduction in cardiovascular risk Risk of adverse effects ↑ (bleeding, infections, disturbances in diabetes control, and gastrointestinal symptoms)	[106]
RAAS inhibitors	-	Experimental model	ApoE−/− mice	Anti-atherogenic effect ↑	[107]
	Ramipril/ perindopril	Clinical trial	Older patients (over 55) at high risk of future cardiovascular complications Patients at lower ASCVD risk with stable coronary artery disease and no clinical evidence of HF	Cardiovascular death ↓ Myocardial infarction and stroke ↓	[108]
Brown adipocyte activation	Cold stimulation	Clinical trial	44 patients with obesity	Adiponectin-to-leptin rate ↑ TG ↓ HDL cholesterol ↑	[109]
	Mirabegron (β3-adrenergic receptor agonists)	Clinical trial	13 sedentary subjects (BMI over 27) 14 healthy women	Insulin resistance ↓ Haemoglobin A1c ↓ Lipolysis ↑ Adiponectin ↑ HDL ↑	[111,112]
Invasive techniques	Bariatric surgery	Cohort study	126 patients with morbid obesity 29 patients with obesity	Blood pressure ↓ PRAT thickness ↓ BMI ↓ LDL cholesterol ↓ and TG ↓	[11,115]

ASCVD—atherosclerotic cardiovascular disease; BMI—body mass index; CRP—C-reactive protein; GLP-1 RAs—glucagon-like peptide-1 receptor agonists; HDL—high-density lipoprotein cholesterol; HF—heart failure; LDL—low-density lipoprotein cholesterol; PPARγ—peroxisome proliferator-activated receptor gamma; PRAT—perirenal adipose tissue; SGLT2—sodium-glucose cotransporter 2; TG—triglyceride; T2DM—type 2 diabetes mellitus; VCAM—vascular cell adhesion molecule 1 ↑—increase; ↓—decrease.

## Data Availability

No new data were created or analysed in this study. Data sharing is not applicable to this article.

## Abbreviations

The following abbreviations are used in this manuscript:

ACAT-1	Acetyl-coenzyme A acetyltransferase 1
ACE	Angiotensin-converting enzyme
ACOT11	Acyl-CoA thioesterase 11
ADMSC	Adipose-derived mesenchymal stem cell
Ang II	Angiotensin II
ASCVD	Atherosclerotic cardiovascular disease
BMI	Body mass index
BMP4	Bone morphogenetic protein 4
CD	Cluster of differentiation
CGRP	Calcitonin gene-related peptide
CLSTN3	Calsyntenin-3
CRP	C-reactive protein
CT	Computed tomography
CTRPs	C1q/TNF-related proteins
CVD	Cardiovascular disease
CYP1B1	Cytochrome P450 1B1 enzyme
eNOS	Endothelial nitric oxide synthase
EAT	Epicardial adipose tissue
Epsti1	Epithelial stromal interaction 1
FAAs	Free fatty acids
FABP3	Fatty acid binding protein 3
FATP-1	Batty acid transport protein 1
ICAM-1	Intracellular adhesion molecule
IL	Interleukin
IRS2	Insulin receptor substrate 2
GLP-1 RAs	Glucagon-like peptide-1 receptor agonists
GLUT4	Glucose transporter type 4
HCA2	Hydroxycarboxylic acid receptor 2
HDL cholesterol	High-density lipoprotein cholesterol
HIF-1 α	Hypoxia-inducible factor 1 α
HOMA-IR	Homeostatic model assessment for insulin resistance
HOXC	Homeobox protein Hox-C
LDL cholesterol	Low-density lipoprotein cholesterol
MCP-1	Monocyte chemotactic protein-1
MMP-9	Matrix metalloproteinase-9
Mpv17l	Mitochondrial inner membrane protein-like 17l
MRI	Magnetic resonance imaging
NADPH oxidase	Nicotinamide adenine dinucleotide phosphate oxidase
NLRP3	NOD-like receptor protein 3
NK cell	Natural killer cell
NO	Nitric oxide
NRF	NF-κB repressing factor
Nrg4	Neuregulin-4
oxLDL	Oxidized LDL
PAD	Peripheral arterial disease
PGC-1α	Peroxisome proliferator-activated receptor gamma coactivator 1-alpha
PGYM	Glycogen phosphorylase, muscle associated
PPARγ	Peroxisome proliferator-activated receptor γ
PRAT	Perirenal adipose tissue
PRAT-SVF	Stromal vascular fraction of perirenal adipose tissue
RAAS	Renin–angiotensin–aldosterone system
ROS	Reactive oxygen species
SR-A1	Class A1 scavenger receptors
SBP	Systolic blood pressure
SCFA	Short-chain fatty acid
SGLT2	Sodium-glucose cotransporter 2
SPARC	Secreted protein acidic and rich in cysteine
Tbx1	T-box protein 1
TG	Triglyceride
TGF-β	Transforming growth factor β
TNF-α	Tumour necrosis factor-α
Tmem26	Transmembrane protein 26
TLRs	Toll-like receptors
TRPV1	Transient receptor potential vanilloid 1
UCP-1	Uncoupling protein 1
VCAM-1	Vascular cell adhesion molecule 1
VEGF-R	Vascular endothelial growth factor receptor
VSMC	Vascular smooth muscle cell
TFAM	Mitochondrial transcription factor A
